# Supplementary material for: Serotonin signaling modulates aging-associated metabolic network integrity in response to nutrient choice in Drosophila melanogaster
Source: Commun Biol. 2021 Jun 15;4:740. doi: 10.1038/s42003-021-02260-5 (PMC8206115; doi:10.1038/s42003-021-02260-5)
Supplement: Supplementary file 2 — Supplementary Information [file 42003_2021_2260_MOESM2_ESM.pdf]

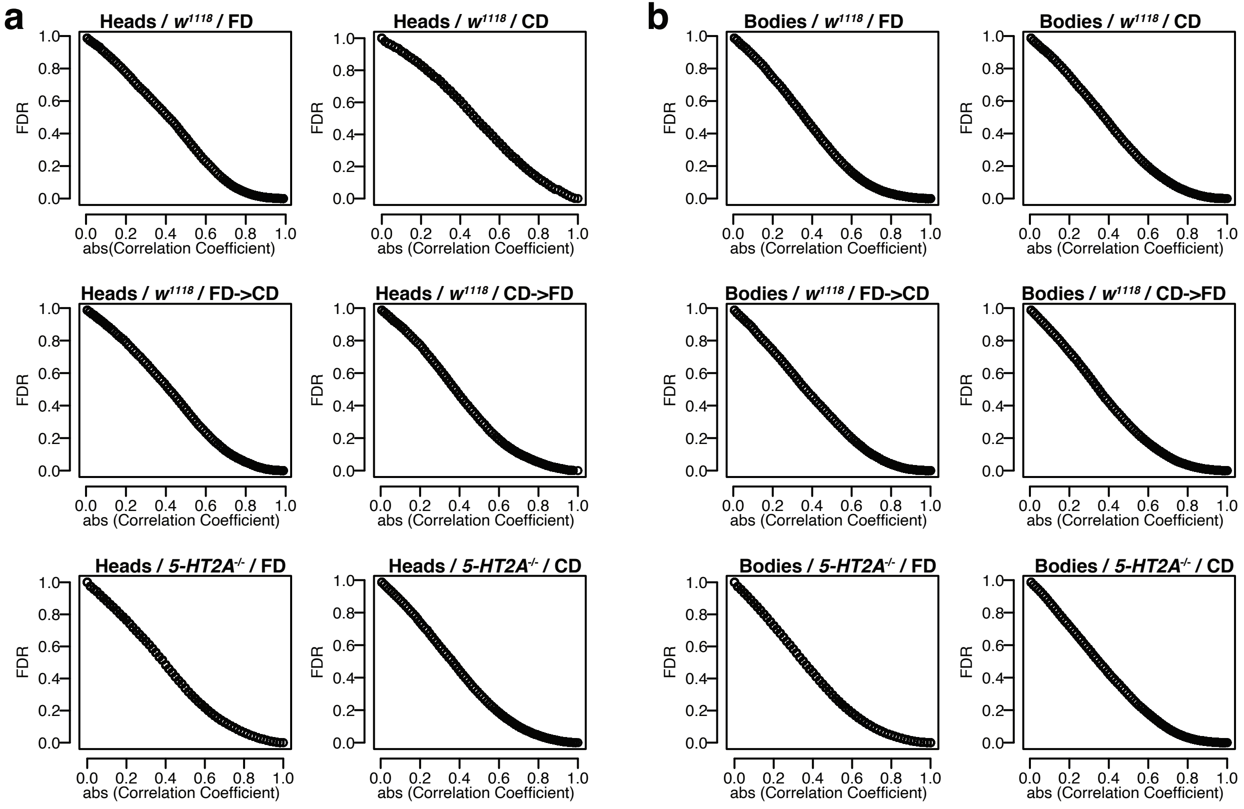

Supplementary Figure 1. Dot plots showing the linear relationship between FDR and the absolute value of correlation coefficient in heads (a) and bodies (b), respectively.

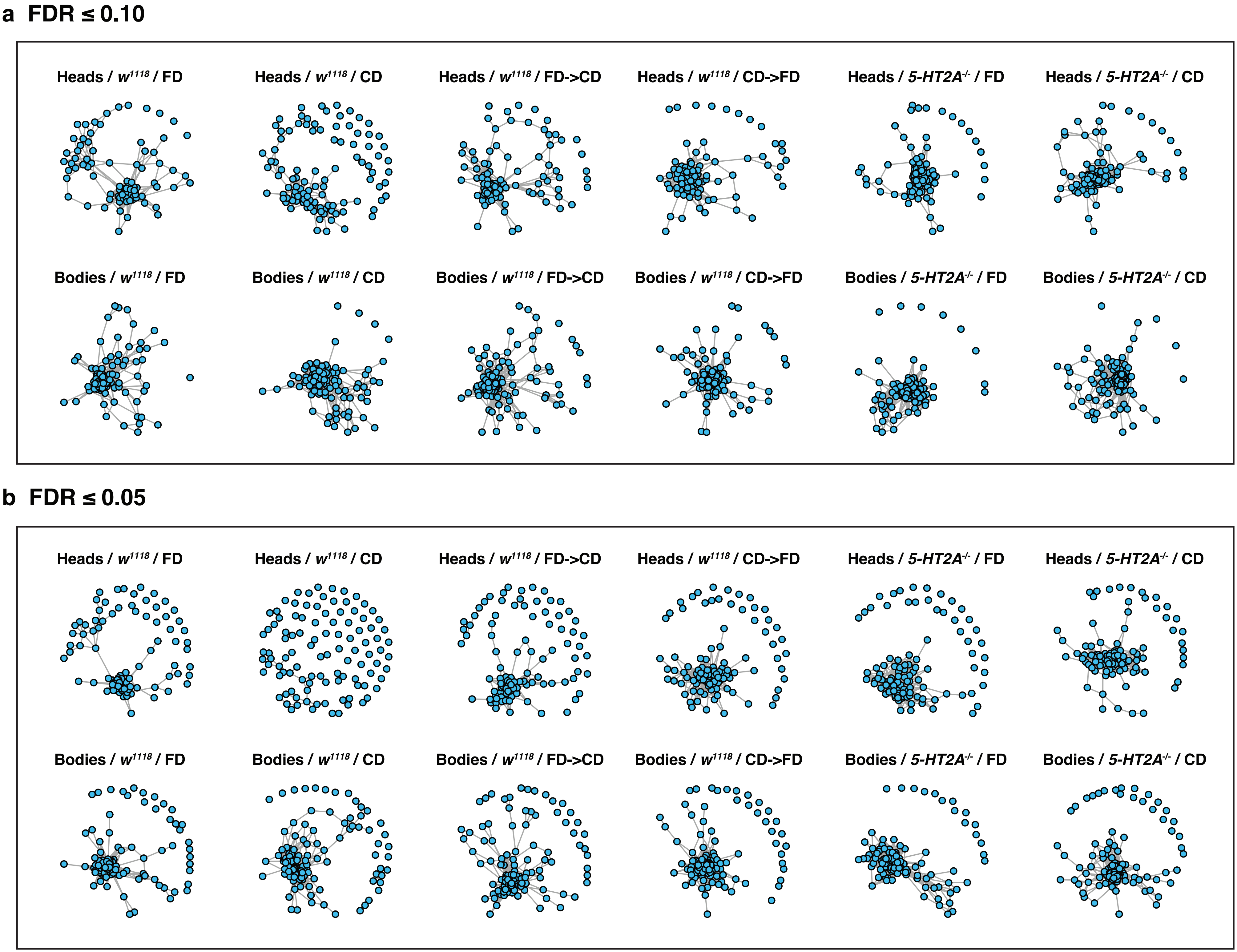

Supplementary Figure 2. Network visualization for each group. The Fruchterman-Reingold algorithm was used to demonstrate network organizations. We applied two cut-offs, FDR ≤ 0.10 (**a**) and FDR ≤ 0.05 (**b**) to determine the edges of correlational networks. As the FDR ≤ 0.05 cut-off generated very few links in the network that was derived from the heads of control flies fed a choice diet, we decided to use FDR ≤ 0.10 for network construction as this criteria yielded similar network structures among groups.

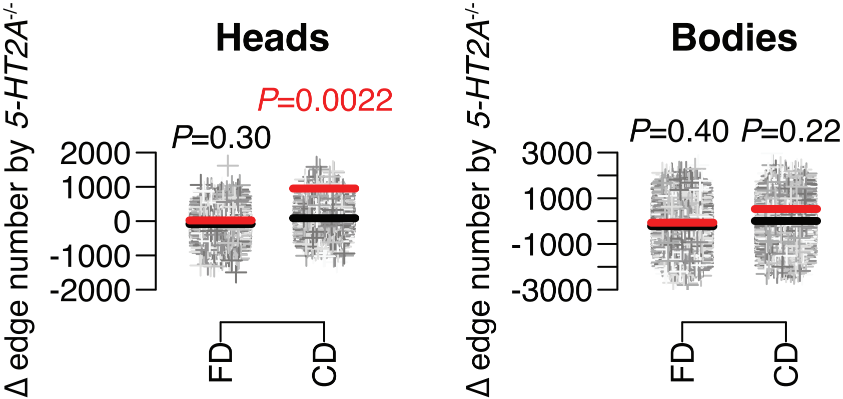

Supplementary Figure 3. Mutated *5-HT2A* allele increases the number of edges in correlation networks in a diet- and tissue-dependent manner. We plotted the edge number of real networks (denoted as the red bar) against randomized networks (as shown by the grey dots, N = 10,000; black bars indicate the median value of each group). *P*-values were obtained by permutation test. The *P*-element insertion in *5-HT2A* significantly increases the number of edges in the heads of flies fed on a choice diet ($P = 0.002$), but not in any other groups ($P > 0.05$).

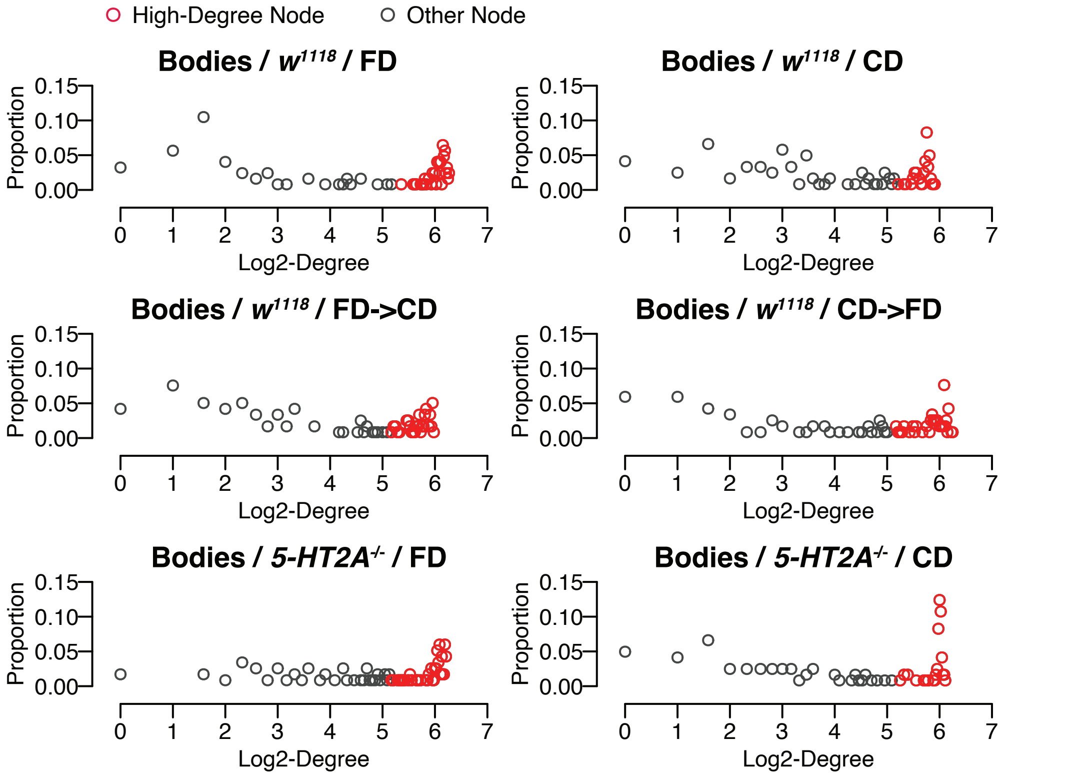

Supplementary Figure 4. Neither dietary switch nor a mutation in *5-HT2A* influences the proportion of high-degree nodes in bodies. Dot plots show the frequency of high-degree nodes and other nodes in each body group. Unlike the results from head groups, neither dietary choice, dietary switch, nor *5-HT2A* mutation affects the high-degree nodes in body networks.

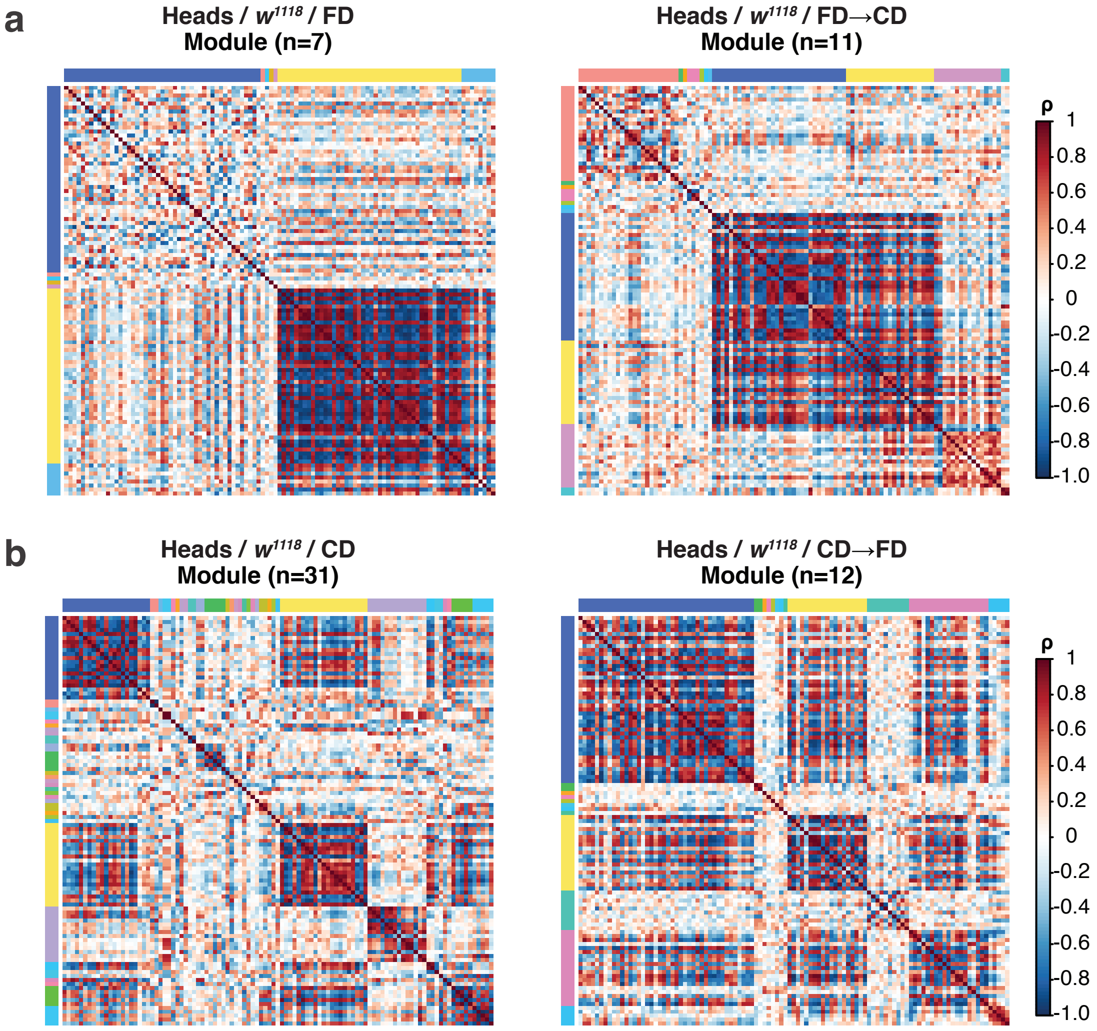

Supplementary Figure 5. Partial reversal of community structures through a 48hr dietary switch. Correlation plots showing the effects of dietary switch on community structures. Color bars on the side/top of the plots indicate individual modules, with yellow and blue bars representing the dominant ones (those who have more metabolites than other modules) in each group. Either switching to a choice diet (**a**) or a fixed diet (**b**) for 48 hrs is sufficient to influence the number and size of network modules, yet the effects are partial as the modularity is in between the unswitched groups.

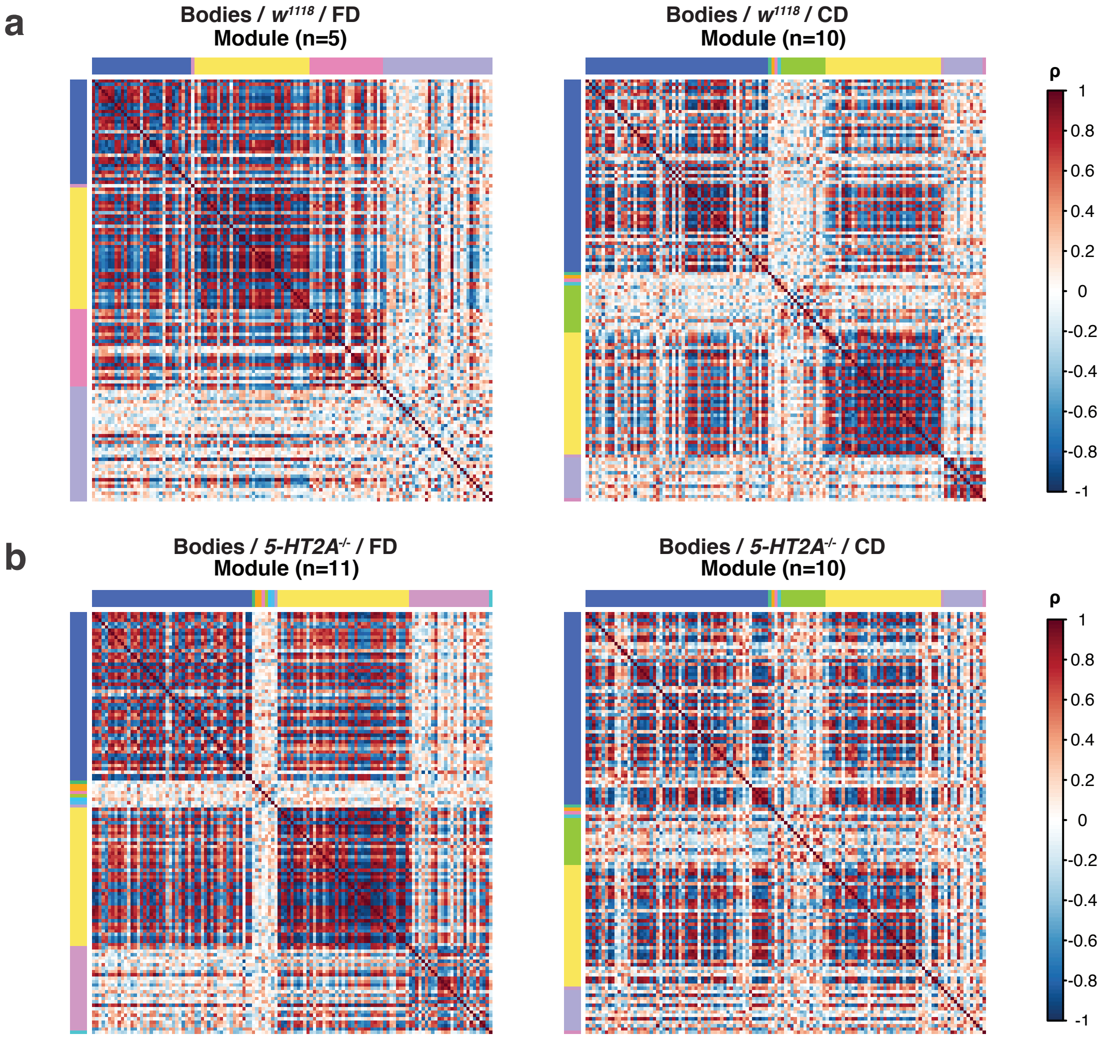

Supplementary Figure 6. Dietary effects on network modularity in bodies. Different from the head results, choice-induced network fragmentation in bodies is milder in control (**a**) and *5-HT2A* mutant (**b**) flies.
