# Supplementary material for: Serotonin signaling modulates aging-associated metabolic network integrity in response to nutrient choice in Drosophila melanogaster
Source: Commun Biol. 2021 Jun 15;4:740. doi: 10.1038/s42003-021-02260-5 (PMC8206115; doi:10.1038/s42003-021-02260-5)
Supplement: Supplementary file 3 — Description of Additional Supplementary Files [file 42003_2021_2260_MOESM3_ESM.pdf]

Description of Additional Supplementary Files

File Name: Supplementary Data 1
Description: Source Data for the starvation resistance analyses.
