# Supplementary material for: Serotonin signaling modulates aging-associated metabolic network integrity in response to nutrient choice in Drosophila melanogaster
Source: Commun Biol. 2021 Jun 15;4:740. doi: 10.1038/s42003-021-02260-5 (PMC8206115; doi:10.1038/s42003-021-02260-5)
Supplement: Supplementary file 6 — Permission Statement [file 42003_2021_2260_MOESM6_ESM.pdf]

Permission Statement

We adapted our previously published image in this manuscript (Figure 1a-d), as the data is crucial to lay the background and motivation of this study to our audiences. The original result is published recently on eLife (2021;10:e59399), and we cited the source of the data and permission statement in the figure legend. According to eLife (https://elifesciences.org/terms), we are free to use and adapt this result as it is under a CC-BY license:

**License to Use Journal Articles and Related Content**

Unless otherwise indicated, the articles and journal content published by eLife on the eLife Sites are licensed under a Creative Commons Attribution license (also known as a CC-BY license). This means that you are free to use, reproduce and distribute the articles and related content (unless otherwise noted), for commercial and noncommercial purposes, subject to citation of the original source in accordance with the CC-BY license.
